# Expression of myogenic, growth related, mitochondrial and antioxidant genes across developmental stage, muscle type, and genotype in Japanese quail skeletal muscle

**DOI:** 10.1016/j.psj.2025.106351

**Published:** 2025-12-28

**Authors:** Gonca Sonmez, Emre Arslan, M. Hudai Culha, Merve Tok

**Affiliations:** a Selcuk University, Faculty of Veterinary Medicine, Department of Genetics, Konya, Turkiye; b Selcuk University, Faculty of Veterinary Medicine, Department of Animal Science, Konya, Turkiye

**Keywords:** Gene expression, Japanese quail, Skeletal muscle, Myogenesis, qPCR

## Abstract

This study examined the developmental and genotype dependent expression of key myogenic (*MYOD1, MYOG, PAX7*), growth related (*IGF1, GHR, MSTN*), mitochondrial (*ANT, COXIII, UCP*), and antioxidant (*SOD1, CAT*) genes in skeletal muscles of Japanese quail (*Coturnix japonica*). Muscle samples from *pectoralis major* (glycolytic) and *biceps femoris* (oxidative) were collected at post hatch days 3 and 42 from three genotypes (Japanese, Jumbo, and Texas). RT-qPCR data, normalized with *ACTB*, revealed distinct stage, muscle, and genotype dependent regulation across gene groups. Early post hatch stages showed elevated expression of *MYOD1, MYOG*, and *IGF1*, associated with active myoblast proliferation, while *MSTN* and *UCP* increased at day 42, reflecting developmental stage–related changes associated with muscle maturation and metabolic adaptation. *ANT* and *COXIII* showed higher expression in biceps femoris, consistent with enhanced oxidative metabolism, whereas *UCP* expression was higher at day 42, predominantly in the pectoralis major. Antioxidant genes exhibited muscle specific expression: *SOD1* and *CAT* were consistently higher in biceps femoris, suggesting stronger redox regulation in oxidative muscle fibers. Several genes also displayed significant interaction effects among developmental stage, muscle type, and genotype, highlighting context dependent regulation of individual gene expression patterns related to muscle growth and metabolism. Overall, these findings describe robust expression differences between the two post hatch stages examined and underscore that muscle type and genetic background contribute to the regulation of myogenic, growth related, mitochondrial, and antioxidant genes in quail skeletal muscle.

## Introduction

The Japanese quail (*Coturnix japonica*) has been widely recognized as a valuable model species in poultry production due to its short generation interval, rapid growth rate, and relatively low maintenance costs. It is particularly preferred for the investigation of economically important traits such as body weight, muscle development, feed efficiency, and carcass characteristics (Minvielle, 2004; Choi et al., 2013; Tavaniello et al., 2014). Differences in growth performance across genetic lines further highlight the quail’s importance as a biological resource in selection studies (Choi et al., 2013; Haqani et al., 2021, 2023). Therefore, quail represents an effective model for exploring genetic and molecular mechanisms underlying muscle development in avian species.

During the early stages of muscle development, myogenic regulatory factors play essential roles. Myogenic differentiation 1 (MYOD1) and myogenin (MYOG) drive myoblast differentiation and myotube formation, whereas paired box 7 (PAX7) ensures the proliferation and maintenance of satellite cells (Relaix et al., 2005; Collins et al., 2005; Chen et al., 2019). The temporal expression profiles of these genes during embryonic and post hatch stages are directly associated with muscle growth and regenerative capacity (Kim et al., 2020, 2021; Choi et al., 2025). Collectively, these myogenic regulators orchestrate early muscle formation and sustain post hatch growth.

Among the endocrine and molecular regulators of muscle growth, insulin like growth factor 1 (IGF1), growth hormone receptor (GHR), and inhibitory regulators such as myostatin (MSTN) are of particular significance. IGF1 and GHR constitute the core of the somatotropic axis, mediating the anabolic actions of growth hormone (Gasparino et al., 2014; Ndunguru et al., 2024). Their expression is known to respond to nutritional and environmental stimuli in poultry species, and multiple studies have demonstrated that their expression is influenced by nutrition, in ovo interventions, and environmental factors. For instance, supplementation with whey protein and Bacillus subtilis enhanced *IGF1* and *GHR* expression, leading to improved growth performance (Jazi et al., 2020). Similarly, in ovo methionine injection or exposure to heat stress altered *IGF1* and *GHR* levels in Japanese quail (Gasparino et al., 2014; Ndunguru et al., 2024). Conversely, MSTN functions as the most potent negative regulator of muscle growth, with elevated expression levels being associated with reduced muscle mass and growth rate (Lee, 2004; Kim et al., 2020). Accordingly, MSTN serves as a critical counter regulatory factor within the group of growth related genes.

Genes involved in energy metabolism are equally crucial for meeting the bioenergetic demands of muscle cells. Cytochrome c oxidase subunit III (COXIII), a key subunit of the mitochondrial respiratory chain, has been identified within the mitochondrial genome of Japanese quail (Nishibori et al., 2001). Adenine nucleotide translocase (ANT) and uncoupling protein (UCP) are essential for ATP transport, energy efficiency, and thermoregulation (Toyomizu et al., 2002; Gasparino et al., 2014). Research has shown that birds with higher feed efficiency exhibit elevated *COXIII* and *ANT* expression, whereas *UCP* expression is upregulated in birds with lower feed efficiency (Gasparino et al., 2014). Moreover, *UCP* expression can be induced in avian skeletal muscle by cold exposure (Toyomizu et al., 2002). Together, these genes illustrate how mitochondrial function contributes to both energy efficiency and environmental adaptation.

Skeletal muscle is constantly exposed to reactive oxygen species generated during metabolic activity, and its protection relies on antioxidant enzymes such as SOD1 and CAT. These enzymes are fundamental for maintaining cellular integrity and muscle health (Aengwanich and Suttajit, 2010). Evidence demonstrates that their expression can be modulated by environmental stressors and dietary factors. For example, dietary supplementation with zinc oxide nanoparticles significantly enhanced antioxidant defense by upregulating *SOD1* and *CAT* expression in Japanese quails (El-Bahr et al., 2020). Conversely, exposure to environmental contaminants such as atrazine disrupted the antioxidant balance by suppressing SOD and CAT activities and inducing oxidative stress in quail liver (Zhang et al., 2017). These observations emphasize the sensitivity of antioxidant responses to physiological and environmental challenges.

Previous research has primarily examined these groups of genes in single tissues, within a single genotype, or under specific environmental conditions. However, no study has yet provided a comprehensive comparison of myogenic, growth related, energy metabolism, and antioxidant genes across distinct muscle types (oxidative biceps femoris vs. glycolytic pectoralis major), quail genotypes (Japanese, Jumbo, and Texas), and developmental stages (day 3 and day 42). Therefore, the present study was designed to simultaneously evaluate these functional gene groups to provide an integrated overview of myogenic, growth related, energy metabolism, and antioxidant gene expression across different muscle types, quail genotypes, and developmental stages.

## Materials and methods

### Experimental design

A total of 36 quail (12 individuals per genotype: Japanese, Jumbo, and Texas) were used in this study. Both sexes were included in the study, and experimental groups were established with an intended balanced representation of males and females. Sex was not treated as an independent experimental factor, as the study was primarily designed to evaluate developmental stage and genotype dependent variation in gene expression. At day 42, sex was anatomically determined at slaughter based on gonadal morphology, whereas at day 3, reliable sex determination was not feasible by gross anatomical examination. Birds were slaughtered at two developmental stages, day 3 (posthatch) and day 42, after body weights were recorded using a calibrated analytical balance (AJ-2200CE, Shinko Denshi Co., Ltd., Tokyo, Japan). At each stage, a total of 36 muscle tissue samples were collected (18 from *musculus biceps femoris* and 18 from *musculus pectoralis major*). In total, 72 muscle samples were obtained, which enabled comparative analyses across developmental stages, muscle types, and genotypes. All birds were reared under identical husbandry and environmental conditions to minimize external variability. The nutrient composition of the basal diet, including proximate components, vitamins, and microelements, is detailed in Supplementary Table S1. Nutrient values were provided by the feed supplier and represent calculated (predicted) values based on ingredient specifications, ensuring standardized nutritional conditions across groups. All experimental procedures were conducted in compliance with institutional and national guidelines for the care and use of laboratory animals and were approved by the Selçuk University Faculty of Veterinary Medicine, Experimental Animal Production and Research Center Ethics Committee (approval no: 2025/73).

### Sample collection and molecular analysis

For molecular analyses, tissue samples from the pectoralis major and biceps femoris were excised, immediately snap frozen in liquid nitrogen, and stored at −80°C until analysis. Total RNA was extracted from ∼50 mg of frozen tissue using TRIzol Reagent (catalog no. 15596026, Invitrogen, Thermo Fisher Scientific, Carlsbad, CA, USA) according to the manufacturer’s protocol. The RNA pellet was washed with ethanol, air dried, and dissolved in DEPC treated ddH₂O. RNA concentration and purity were determined spectrophotometrically using a colibri spectrometer (Titertek Berthold, Berthold Detection Systems GmbH, Pforzheim, Germany). RNA quality was evaluated based on A260/280 ratio (≥ 1.8). Genomic DNA was removed by DNase I treatment (catalog no. EN0521, Thermo Fisher Scientific, Waltham, MA, USA) and cDNA was synthesized from 1 µg of RNA using the iScript™ cDNA Synthesis Kit (catalog no. 170-8891, Bio-Rad Laboratories, Hercules, CA, USA). Quantitative PCR (qPCR) was performed using the SsoAdvanced™ Universal SYBR® Green Supermix (catalog no. 172-5271, Bio-Rad Laboratories, Hercules, CA, USA) on a LightCycler® Nano System 1.0 (Roche Diagnostics GmbH, Mannheim, Germany). Cycling conditions consisted of an initial denaturation at 95°C for 3 min, followed by 45 cycles of denaturation at 95°C for 15 s, annealing at the gene specific temperature for 30 s, and extension at 72°C for 30 s. Specificity was verified by melting curve analysis performed at the end of each run. No template controls were included in each run to verify the absence of contamination or nonspecific amplification. Six biological replicates were analyzed per group, and each reaction was run in technical triplicate. Primer sequences, NCBI accession numbers, amplicon sizes, annealing temperatures, amplification efficiencies (90–110%), and references for previously published primers are provided in Supplementary Table S2.

### Statistical analysis

All statistical analyses were performed using IBM SPSS Statistics v22.0 (IBM Corp., Armonk, NY, USA). A 3 × 2 × 2 factorial design was applied, corresponding to three genotypes (Japanese, Jumbo, and Texas), two muscles (pectoralis major and biceps femoris), and two developmental stages (days 3 and 42), with six biological replicates per combination. Sex was not included as a factor in the statistical model, as the study was not designed to test sex specific effects and reliable sex determination was not feasible at day 3. Each qPCR reaction was performed in triplicate technical replicates. ΔCt values were used for all statistical analyses, whereas 2^^−ΔΔCt^ values were used only for fold change visualization in figures and tables. Relative expression levels were calculated using the 2^^−ΔΔCt^ method (Livak and Schmittgen, 2001) with *ACTB* (β-actin) as the reference gene (Carvalho et al., 2019). For relative quantification, the day 3 biceps femoris group, which exhibited the highest mean Ct values (indicating the lowest baseline expression), was selected as the control group, and all other expression levels were normalized relative to this control. Results are expressed as mean ± SEM. Data normality was assessed with the Shapiro–Wilk test, and homogeneity of variances with Levene’s test. Parametric data were expressed as mean ± SEM, whereas non parametric data were presented as median (interquartile range, IQR). For body weight data, Welch’s ANOVA followed by Games–Howell post hoc tests was applied when variance assumptions were violated.

Gene expression data were analysed using statistical models appropriate to their distributional properties. Normally distributed and homogeneous genes (*MYOD1, MYOG, PAX7, IGF1, MSTN, SOD1, CAT*) were evaluated with three way ANOVA (Day × Muscle × Genotype). Significant ANOVA effects were further analyzed using Tukey’s HSD post hoc test. For *GHR*, violated the homogeneity assumption, Welch’s ANOVA with robust standard errors (HC3 correction) was applied. Genes not meeting normality assumptions (*COXIII, UCP, ANT*) were analysed using a nonparametric aligned rank transform (ART) ANOVA implemented in R (v4.5.1), which enables factorial testing of interaction effects before the evaluation of main effects.

## Results

Slaughter body weights measured at day 3 and day 42 did not differ significantly among the three genotypes (Table 1). Welch ANOVA confirmed the absence of genotype effects at day 3 (F(2, 9.78) = 1.07, *P* = 0.38) and day 42 (F(2, 6.92) = 1.18, *P* = 0.36). Where significant higher-order interactions were detected, only interaction effects are reported.

**Table 1:** Body weight at slaughter (mean ± SD) of Japanese, Jumbo, and Texas quails at day 3 and day 42.

Genotype	Day 3 (g) Mean ± SD	Day 42 (g) Mean ± SD
Japanese	9.57 ± 0.81	154.83 ± 2.52
Jumbo	9.67 ± 0.70	166.30 ± 17.32
Texas	8.92 ± 1.03	154.65 ± 18.81

### Myogenic genes (MYOD1, MYOG, PAX7)

**Fig. 1:**
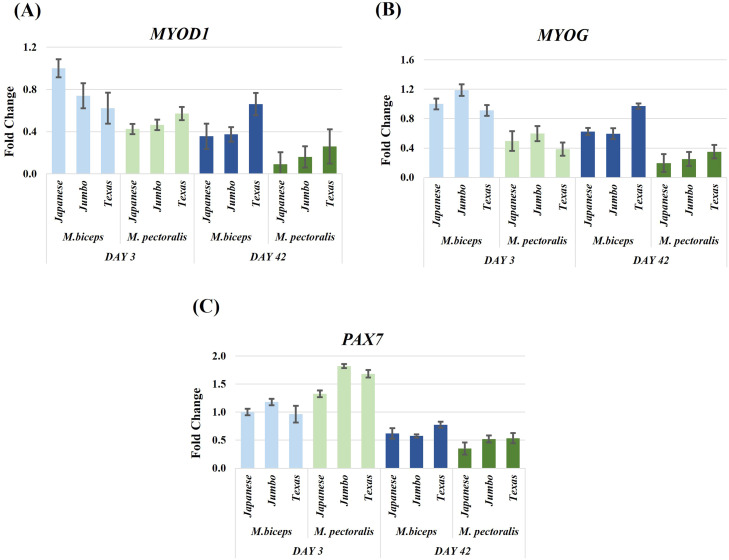
Relative expression levels of myogenesis related genes (*MYOD1, MYOG, PAX7*) in biceps femoris and pectoralis major muscles of Japanese quail at day 3 and day 42. Expression was analyzed across three genotypes (Japanese, Jumbo, Texas). *ACTB* was used as the reference gene and the Japanese biceps femoris at day 3 served as the control group. Data are presented as fold change ± SEM.

Expression of myogenic regulatory genes showed several significant interaction effects involving developmental stage, muscle type, and genotype (Fig. 1, Table 2, Supplementary Table S3). *MYOD1* expression showed significant Day × MuscleType (F(1,60) = 4.38, *P* = 0.04, partial η² = 0.07) and Day × Genotype interactions (F(2,60) = 3.72, *P* = 0.03, partial η² = 0.11). *MYOG* expression exhibited a significant Day × Genotype interaction (F(2,60) = 2.18, *P* = 0.02, partial η² = 0.12). *PAX7* expression was strongly influenced by a Day × MuscleType interaction (F(1,60) = 13.51, *P* < 0.001, partial η² = 0.18), with differences observed between muscles across developmental stages.

**Table 2:** Summary of statistical significance (P values) for main and interaction effects in gene expression analyses.

Gene	Day	Muscle Type	Genotype	Day x Muscle	Day x Genotype	Muscle x Genotype	3 Way
MYOD1	***	***	ns	*	*	ns	ns
*MYOG*	***	***	ns	ns	*	ns	ns
*PAX7*	***	ns	ns	***	ns	ns	ns
*IGF1*	***	ns	*	***	*	ns	ns
*GHR*	ns	***	*	***	ns	ns	*
*MSTN*	***	***	***	*	ns	ns	ns
*ANT*	***	***	***	ns	ns	ns	ns
*COXIII*	***	***	ns	***	ns	ns	*
*UCP*	***	ns	ns	***	**	ns	ns
*SOD1*	*	***	ns	ns	ns	ns	ns
*CAT*	ns	***	*	***	*	ns	ns

Genes were analysed by three way ANOVA or aligned rank transform (ART) ANOVA as appropriate. Parametric ANOVA results are provided in Supplementary Table S3, while ART-ANOVA results for non-parametric data (*ANT, COXIII*, and *UCP*) are reported in Supplementary Table S4. Significance levels are shown as *P ≤ 0.05, **P ≤ 0.01, ***P ≤ 0.001; ns = not significant.

### Growth related genes (IGF1, GHR, MSTN)

**Fig. 2:**
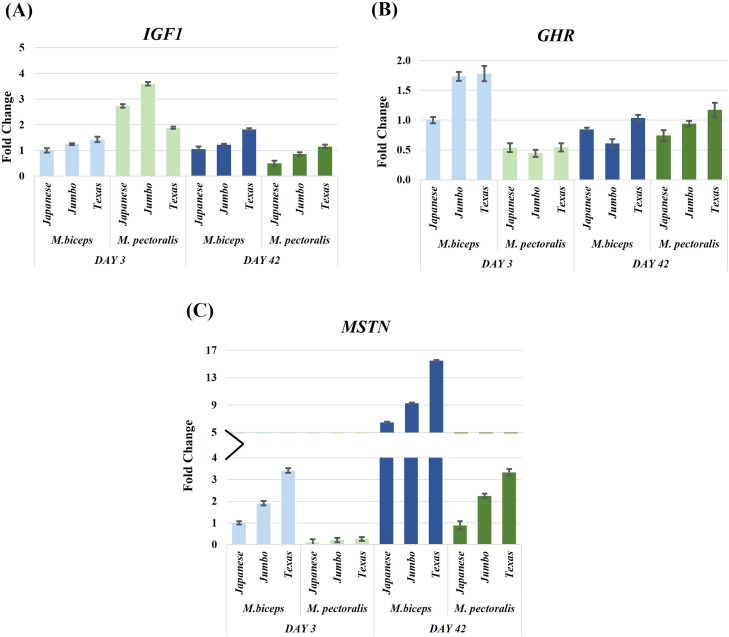
Relative expression levels of growth related genes (*IGF1, GHR, MSTN*) in M. biceps femoris and M. pectoralis major muscles of Japanese quail at day 3 and day 42. Expression patterns were assessed across three genotypes (Japanese, Jumbo, Texas). *ACTB* served as the reference gene and the Japanese biceps femoris at day 3 served as the control group. Data are shown as fold change ± SEM. The y axis for *MSTN* (panel C) is broken to better illustrate large differences in expression levels.

Several significant interaction effects involving developmental stage, muscle type, and genotype were detected for growth related genes (Fig. 2, Table 2, Supplementary Table S3). *IGF1* expression showed significant Day × MuscleType (F(1,60) = 43.08, *P* < 0.001, partial η² = 0.42) and Day × Genotype interactions (F(2,60) = 4.91, *P* = 0.01, partial η² = 0.14). *GHR* expression was influenced by a significant Day × MuscleType interaction (F(1,60) = 33.73, *P* < 0.001, partial η² = 0.36) as well as a three way Day × MuscleType × Genotype interaction (F(2,60) = 3.42, *P* = 0.04, partial η² = 0.10). *MSTN* expression exhibited a significant Day × MuscleType interaction (F(1,60) = 4.77, *P* = 0.03, partial η² = 0.07).

### Mitochondrial Genes (ANT, COXIII, UCP)

**Fig. 3:**
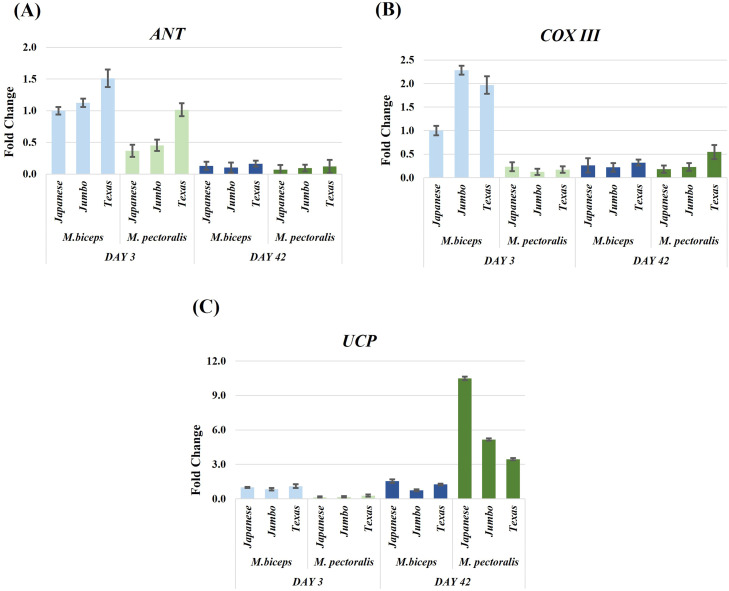
Relative expression levels of mitochondrial energy metabolism related genes (*ANT, COXIII, UCP*) in M. biceps femoris and M. pectoralis major muscles of Japanese quail at day 3 and day 42. Analyses included three genotypes (Japanese, Jumbo, Texas). *ACTB* was used as the reference gene and the Japanese biceps femoris at day 3 served as the control group. Data are presented as fold change ± SEM.

Mitochondrial gene expression data were analysed using a non parametric aligned rank transform (ART) ANOVA due to violations of normality assumptions (Fig. 3, Table 2, Supplementary Table S4). *ANT* expression showed significant main effects of developmental stage (F₁,₆₀ = 185.45, *P* < 0.001), muscle type (F₁,₆₀ = 27.15, *P* < 0.001), and genotype (F₂,₆₀ = 8.67, *P* < 0.001). No significant two way or three way interaction effects were detected (Supplementary Table S4). *COXIII* expression was characterised by significant Day × MuscleType (F₁,₆₀ = 79.98, *P* < 0.001) and Day × MuscleType × Genotype interactions (F₂,₆₀ = 3.64, *P* = 0.032). *UCP* expression displayed significant Day × MuscleType (F₁,₆₀ = 164.78, *P* < 0.001) and Day × Genotype interactions (F₂,₆₀ = 5.97, *P* = 0.004).

### Antioxidant genes (SOD1, CAT)

**Fig. 4:**
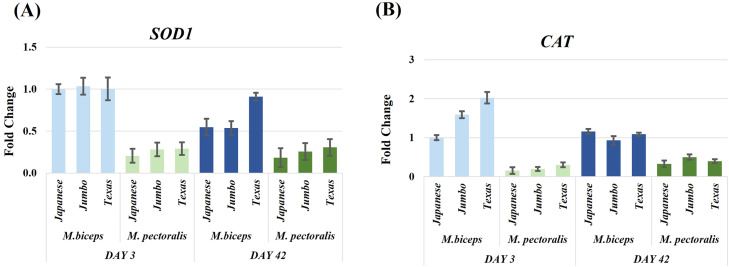
Relative expression levels of antioxidant related genes (*SOD1, CAT*) in M. biceps femoris and M. pectoralis major muscles of Japanese quail at day 3 and day 42. Expression was evaluated in three genotypes (Japanese, Jumbo, Texas). *ACTB* was used as the reference gene, and the Japanese biceps femoris at day 3 served as the control group. Data are presented as fold change ± SEM.

*SOD1* expression was significantly affected by developmental stage (F(1,60) = 4.27, *P* = 0.04) and muscle type (F(1,60) = 91.56, *P* < 0.001). No significant genotype or interaction effects were detected (Fig. 4A, Table 2, Supplementary Table S3). *CAT* expression exhibited significant Day × MuscleType (F(1,60) = 24.00, *P* < 0.001) and Day × Genotype interactions (F(2,60) = 3.25, *P* = 0.05) (Fig. 4B, Table 2, Supplementary Table S3).

## Discussion

Myogenic regulatory factors (MYOD1, MYOG, and PAX7) are key transcriptional regulators that orchestrate the sequential stages of skeletal muscle formation and maintenance (Relaix et al., 2005; Collins et al., 2005; Choi et al., 2014). Among them, MYOD1 governs the commitment of precursor cells and the initiation of myoblast proliferation, MYOG controls the terminal differentiation required for myotube formation, and PAX7 ensures the preservation of the satellite cell population throughout muscle development (Relaix et al., 2005; Choi et al., 2014). In the present study, *MYOD1* and *MYOG* transcripts were highly expressed at day 3 but markedly declined by day 42, indicating higher myogenic gene expression during the early post hatch stage. A similar developmental pattern was described by Shin et al. (2009). The relatively high *PAX7* expression in the pectoralis major at day 3 is consistent with an active satellite cell pool during early development.

IGF1 plays a central role in somatotropic growth regulation (Lee, 2004; El Sabry et al., 2017). In the present study, *IGF1* expression was higher at day 3 than at day 42, indicating relatively higher *IGF1* transcript abundance during the early post hatch stage. Muscle specific differences were observed, suggesting muscle type dependent expression patterns between glycolytic and oxidative muscles (Tavaniello et al., 2014; Ndunguru et al., 2024). Because intermediate developmental stages were not sampled, these findings should be interpreted as descriptive rather than indicative of a defined temporal transition in growth regulation. The regulation of mitochondrial activity is fundamental for sustaining energy metabolism during muscle development (Toyomizu et al., 2002; Choi et al., 2013). *ANT* expression was higher at day 3, consistent with elevated mitochondrial gene expression during early post hatch growth. *GHR* expression exhibited significant muscle and genotype dependent variation, but did not show a uniform developmental pattern across the stages examined. *MSTN* expression was low at day 3 and increased at day 42, consistent with previously reported stage dependent changes in MSTN transcription during muscle maturation (Gasparino et al., 2014). Taken together, the observed expression patterns of *IGF1, GHR*, and *MSTN* highlight stage, muscle, and genotype dependent regulation of growth related genes.

The regulation of mitochondrial activity is fundamental for sustaining energy metabolism during muscle development. Mitochondria generate ATP through oxidative phosphorylation, and the transcription of mitochondrial genes often varies according to developmental stage, muscle type, and metabolic demand (Toyomizu et al., 2002; Choi et al., 2013; Tavaniello et al., 2014). In the present study, *ANT* expression was higher at day 3 compared with day 42, indicating higher *ANT* transcript abundance during the early post hatch stage, as previously reported in avian muscles (Toyomizu et al., 2002). Although genotype effects were detected for *ANT* expression, these differences were not associated with significant interaction terms and therefore are interpreted descriptively rather than as genotype specific developmental patterns. The higher *ANT* expression detected in the biceps femoris is consistent with the oxidative characteristics of this muscle, in agreement with Tavaniello et al. (2014), who found increased mitochondrial gene activity in oxidative muscles. *COXIII* expression also differed between early and later developmental stages; however, the magnitude and direction of these changes varied according to muscle type and genotype, reflecting context dependent expression differences rather than implying coordinated or interactive regulation of respiratory chain activity during muscle maturation. Age related modulation of mitochondrial gene expression has likewise been reported in avian species (Toyomizu et al., 2002), and differences between oxidative and glycolytic muscles have been associated with variation in mitochondrial density and metabolic demand (Tavaniello et al., 2014). In contrast, *UCP* expression was low at day 3 and higher at day 42, with this increase being predominantly observed in the pectoralis major. Previous studies have shown that UCP expression rises with age or under environmental stress conditions (Choi et al., 2013; Aengwanich and Suttajit, 2010), suggesting that *UCP* transcription is primarily associated with developmental stage and muscle specific metabolic context rather than genetic background. Overall, mitochondrial gene expression differed between developmental stages and muscle types at the time points examined, indicating stage and muscle dependent variation in mitochondrial transcriptional profiles during post hatch muscle development.

Maintaining oxidative balance during muscle growth is essential for supporting cellular differentiation and sustaining energy metabolism. Therefore, the developmental and tissue specific regulation of antioxidant enzymes such as SOD1 and CAT has been widely used to describe metabolic differences among muscle types (El-Bahr et al., 2020; Zhang et al., 2017). In the present study, *SOD1* expression declined markedly from day 3 to day 42, indicating lower *SOD1* transcript abundance at the later post hatch stage. This reduction was more pronounced in the biceps femoris, which is consistent with its oxidative metabolic profile. The higher *SOD1* transcript abundance in the biceps femoris compared with the pectoralis major is consistent with previous reports describing elevated antioxidant gene expression in oxidative muscles (Zhang et al., 2017; El-Bahr et al., 2020). *CAT* expression did not show a significant developmental change but exhibited pronounced differences between muscles and genotypes. The significantly higher *CAT* expression in the biceps femoris is consistent with muscle type dependent variation in antioxidant gene expression (El-Bahr et al., 2020). Significant Day × Muscle and Day × Genotype interactions confirmed that *CAT* regulation varied dynamically with both tissue type and genetic background, indicating context dependent regulation of *CAT* transcription across developmental stages. Collectively, these findings demonstrate that *SOD1* and *CAT* expression varies according to developmental stage and muscle phenotype in Japanese quail. Taken together, the observed expression patterns indicate stage and muscle dependent variation in antioxidant gene transcription, without implying coordinated enzymatic regulation or causal links with mitochondrial function.

Several limitations of the present study should be acknowledged. Gene expression was assessed at only two post hatch time points (days 3 and 42), limiting inference regarding intermediate developmental stages. In addition, individual pectoralis major and biceps femoris muscle weights were not recorded, preventing direct evaluation of muscle accretion independent of body weight. Although both sexes were included, sex was not incorporated as a factor because reliable sex determination was not feasible at day 3.

## Conclusion

Overall, our results demonstrate clear differences in gene expression between early (day 3) and later (day 42) post hatch stages in *Coturnix japonica* skeletal muscle. In summary, this study describes stage specific expression differences across myogenic (*MYOD1, MYOG, PAX7*), growth regulatory (*IGF1, GHR, MSTN*), mitochondrial (*ANT, COXIII, UCP*), and antioxidant (*SOD1, CAT*) pathways in Japanese quail skeletal muscles. The early post hatch stage was dominated by myogenic activation and anabolic signalling, reflecting stage dependent physiological changes during muscle maturation rather than a defined mechanistic transition. Early post hatch upregulation of *MYOD1, MYOG* and *IGF1*, together with muscle and genotype specific patterns in *PAX7, GHR* and *ANT*, suggests that the timing of myogenic and metabolic gene expression is associated with subsequent muscle growth potential, without implying direct causal regulation. Conversely, late increases in *MSTN* and *UCP* are consistent with developmental stage related shifts toward growth regulation and metabolic adaptation, and should be interpreted as descriptive rather than indicative of growth restraint mechanisms. From a breeding perspective, these profiles identify early stage transcriptional candidates, notably high *MYOD1, MYOG* and *IGF1* expression supported by *PAX7* and *ANT* in the early post hatch period, as potential indicators associated with muscle accretion potential. However, these associations are descriptive and require further validation before application in selection programs. Methodologically, normalization using *ACTB* supports the robustness of these inferences. Future work should (i) validate the predictive value of the proposed early markers across diverse lines and environments, (ii) combine expression markers with genomic breeding values, and (iii) monitor correlated responses in meat quality, thermotolerance and welfare. Collectively, our findings provide a biologically informed framework and a set of candidate molecular markers for understanding post hatch muscle gene expression dynamics in *Coturnix japonica*, rather than implying a defined mechanistic model or an immediately deployable biomarker panel.

## CRediT authorship contribution statement

**Gonca Sonmez:** Writing – original draft, Visualization, Validation, Supervision, Software, Project administration, Methodology, Funding acquisition, Formal analysis, Data curation, Conceptualization. **Emre Arslan:** Writing – review & editing, Resources, Investigation. **M. Hudai Culha:** Methodology, Investigation, Data curation. **Merve Tok:** Resources.

## Disclosures

The authors declare that they have no known competing financial interests or personal relationships that could have appeared to influence the work reported in this paper.

## Appendix Supplementary materials

Image, application 1

Image, application 2

Image, application 3

Image, application 4

Image, application 5
