# Supplementary material for: Expression of myogenic, growth related, mitochondrial and antioxidant genes across developmental stage, muscle type, and genotype in Japanese quail skeletal muscle
Source: Poult Sci. 2025 Dec 28;105(2):106351. doi: 10.1016/j.psj.2025.106351 (PMC12804387; doi:10.1016/j.psj.2025.106351)
Supplement: Supplementary file 1 [file mmc1.docx]

**Supplementary Information**

**Supplementary Table S1.** Nutrient composition of the experimental diet provided to Japanese quail

This table presents the proximate composition, macroelements, vitamins, and microelements included in the diet. Nutrient values were provided by the feed supplier and represent calculated (predicted) values based on ingredient specifications. Values are expressed as percentages or concentrations of each component.

**Supplementary Table S2.** Primer sequences and amplification parameters for target and reference genes

Forward and reverse primer sequences, NCBI accession numbers, amplicon length (bp), annealing temperatures, and references are listed for each gene. These primers were used in qPCR analysis to evaluate gene expression profiles.

**Supplementary Table S3.** Results of General Linear Model (GLM) analysis for the effects of developmental stage, muscle type, and genotype on gene expression

Values represent F statistics, degrees of freedom (df), P values, and effect sizes (Partial η²) for each main effect and interaction term. Significance levels are denoted as follows: ns = not significant (P ≥ 0.05), * = P < 0.05, ** = P < 0.01, *** = P < 0.001.

**Supplementary Table S4.** Results of non parametric aligned rank transform (ART) analysis for the effects of developmental stage, muscle type, and genotype on gene expression levels (*ANT*, *COXIII*, and *UCP*). Values represent F statistics, degrees of freedom (df), and P values obtained from Type III ART ANOVA for each main effect and interaction term.
